# Fluorescein versus Indocyanine Green Angiography Guided Half-Dose Photodynamic Therapy for Chronic Central Serous Chorioretinopathy

**DOI:** 10.18502/jovr.v19i1.15420

**Published:** 2024-03-14

**Authors:** Farzan Moodi, Masood Naseripour, Amin Zand, Reza Mirshahi, Vihan Moodi, Sayyed Amirpooya Alemzadeh, Khalil Ghasemi Falavarjani

**Affiliations:** ^1^Eye Research Center and Department of Ophthalmology, The Five Senses Health Institute, Rassoul Akram Hospital, School of Medicine, Iran University of Medical Sciences, Tehran, Iran; ^2^Stem Cell and Regenerative Medicine Research Center, Iran University of Medical Sciences, Tehran, Iran; ^3^School of Medicine, Tehran University of Medical Sciences, Tehran, Iran

**Keywords:** Central Serous Chorioretinopathy, Fluorescein Angiography, Indocyanine Green Angiography, Photodynamic Therapy

## Abstract

**Purpose:**

To compare the outcomes of fluorescein angiography (FA)-guided and indocyanine green angiography (ICGA)-guided half-dose photodynamic therapy (PDT) in patients with chronic central serous chorioretinopathy (CSC).

**Methods:**

In this retrospective comparative study, medical records of eyes with chronic CSC who underwent half-dose PDT were reviewed. A retina specialist performed FA-guided half-dose PDT, and the other performed ICGA-guided treatment. The success of applying PDT in the resolution of subretinal fluid was compared between the FA- and ICGA-guided methods.

**Results:**

Eighty-two eyes of 73 patients (41 eyes in each group) received half-dose PDT. After half-dose PDT, a significant improvement in the best-corrected visual acuity (BCVA) was found at the time of the last follow-up in both groups (both *P* < 0.001), with no significant intergroup difference. Central subfield and subfoveal choroidal thicknesses decreased significantly in both groups at the last follow-up (all *P* < 0.05), with no significant differences between the groups. Subretinal fluid (SRF) resolved in all eyes, and no persistent SRF was detected during the follow-up period.

**Conclusion:**

FA-guided and ICG-guided half-dose PDT may have similar efficacy for the treatment of chronic CSC.

##  INTRODUCTION

Central serous chorioretinopathy (CSC) is characterized by neurosensory retinal detachment typically localized to the posterior pole. The pathogenesis of CSC is not well-known; however, it is caused by fluid leakage which originates from choriocapillaris and accumulates in the subretinal space through the retinal pigment epithelium (RPE).^[1,2,3]^ Although patients with acute CSC commonly recover without intervention, 30–50% of cases become chronic or persistent.^[4]^

Photodynamic therapy (PDT) is currently considered the treatment of choice for chronic CSC. Standard, full-dose, full-fluence PDT may have some serious complications, including choriocapillaris ischemia, RPE atrophy, and choroidal neovascularization (CNV).^[5]^ Therefore, less invasive PDT techniques, including half-dose and half-fluence PDT, have gained increasing popularity. Half-dose verteporfin (3 mg/m^2^) PDT has shown promising results in the treatment of CSC with a lower risk of complications compared to the standard PDT.^[6,7,8]^ Fluorescein angiography (FA) is the first step in diagnosing CSC and identifying the leakage site. However, treatment with PDT is classically performed on the hyper-permeability areas based on the results of indocyanine green angiography (ICGA) imaging. ICGA absorbs and emits light waves in the near-infrared range. In addition, ICGA diffuses less through the choroidal vasculature. In FA, the light absorption and emission occurs at lower wave lenghts and fluorescence rapidly leaks through the choriocapillaris. Therefore, ICGA is a more appropriate modality for the visualization of choroidal vascular abnormalities, such as hyper-permeability, filling delay, and venous congestion areas. However, ICGA is expensive and time-consuming, and the required device is less available than the device that executes FA.^[9]^ Limited studies have suggested similar efficacy for these two imaging modalities to guide PDT in patients with CSC.^[10,11]^ This study was performed to compare the outcomes of ICGA-guided half-dose PDT with those of FA-guided half-dose PDT in patients with chronic CSC.

##  METHODS

This was a retrospective comparative study on patients who underwent half-dose PDT for the treatment of chronic CSC from December 2016 to December 2021 in Markazi Eye Clinic, Tehran. The study protocol was approved by the Ethical Committee of Iran University of Medical Sciences (IR.IUMS.REC.1400.429). Informed consent was obtained from the patients for the PDT treatment. Patients with neurosensory retinal detachment detected in optical coherence tomography (OCT) lasting more than three months after the onset of the symptoms or those with neurosensory retinal detachment associated with signs of chronic chorioretinopathy, including RPE atrophy in OCT, were included in the study. The exclusion criteria were any evidence of current or previous CNV, other ocular diseases that might affect visual acuity, and a history of previous PDT treatment for CSC. Initially, records of all patients who underwent half dose PDT for chronic CSC were collected. Patients without follow-up were excluded. The ICGA-guided group consisted of 41 patients. Next, to ensure a balanced comparison, 41 cases were randomly selected (keeping the same male/female ratio) from the FA-guided PDT group to match the number of cases in the ICGA group. One of the retina specialists (MN) routinely performed ICGA-guided PDT. FA-guided treatment was performed routinely by another retina specialist (KGF). A Spectralis OCT system (Heidelberg Spectralis OCT; Heidelberg Engineering GmbH, Heidelberg, Germany) was used for retinal and choroidal imaging. FA and ICGA were performed using a Spectralis device (Heidelberg HRA2; Heidelberg Engineering GmbH, Heidelberg, Germany). All OCT, FA, and ICGA images were retrieved from the clinic's computer system. OCT was performed before and one month after PDT. If residual subretinal fluid (SRF) was detected at one-month follow-up, the OCT examination was repeated bimonthly until complete resolution was observed. Follow-up schedules were one month, three months, six months, and the last available visit. OCT scans taken within a scheduled appointment were classified as part of the same follow-up point. The protocol for half-dose PDT has been described elsewhere.^[8]^ Briefly, patients received an infusion of 3 mg/m^2^ of verteporfin (VisudyneⓇ; Novartis, Basel, Switzerland) within 8 min. The laser treatment was then performed 10 min after the start of the infusion with a standard light intensity of 600 mW/cm^2^ and an irradiation time of 83 seconds. The area of laser irradiation was based on FA or ICGA findings. In the FA-guided PDT group, the spot diameter was calculated as 1000 µm surrounding the leaking spots. In the ICGA-guided PDT group, the spot diameter was calculated as 500 µm surrounding the area of choroidal vascular hyperpermeability. If a hyperpermeability was observed outside the macula, it was also treated as much as possible. The comparisons between the two groups were made with respect to baseline characteristics and outcome of treatment. The collected baseline variables were age, sex, foveal involvement, symptom duration, best-corrected visual acuity (BCVA), central subfield thickness (CST), subfoveal choroidal thickness (SCT), PDT spot size, and PDT spot numbers. The main outcome measure was the resolution of SRF. Secondary outcomes were BCVA, CST, and SCT. CST was automatically generated by the OCT device. SCT was manually measured in the foveal center, from the outer surface of RPE to the inner surface of the sclera. Persistence of SRF was defined as no or incomplete resorption of SRF within three months after PDT.^[11]^

### Statistical Analysis

Data were analyzed using the SPSS software version 24 (SPSS Inc., Chicago, IL). The normality of the data was evaluated using the Kolmogorov–Smirnov test, which indicated that the normality assumption was not met. Therefore, nonparametric tests were used for the remaining analyses. Fisher exact test was used to assess categorical cross-distributions. Wilcoxon signed-rank test was performed to compare within groups. In addition, the Mann–Whitney U test and general linear model repeated measure analysis were used to compare between groups. A *P*-value < 0.05 was considered statistically significant.

##  RESULTS

This study included a total of 82 eyes of 73 patients (41 eyes in each group). The baseline characteristics are shown in Table 1. Except for the duration of CSC symptoms before PDT, there were no significant differences between the two groups. The duration of symptoms was significantly longer in the ICGA-guided group (18.14 ± 24.05 months) than in the FA-guided group (6.42 ± 8.80 months); (*P* = 0.03). Patients were followed for a mean of 6.36 ± 6.32 months in the FA-guided PDT group and 6.96 ± 5.82 months in the ICGA-guided PDT group (*P* = 0.55).

BCVA significantly improved at the last follow-up examination after PDT in both groups (both *P* < 0.001). There was no significant difference in the improvement of BCVA between the groups (*P* = 0.70) [Table 2]. SRF resolved completely in all cases, with no significant difference in duration of resorption between the groups (43.76 ± 10.29 days vs 49.94 ± 16.01 days for ICGA- and FA-guided groups, respectively, *P* = 0.10). CST decreased significantly at the last follow-up visit in both groups (both *P* < 0.001). There was no significant difference in the CST reduction between the groups (*P* = 0.56). In addition, SCT decreased significantly at the last follow-up, in both groups (*P* < 0.001 in the FA-guided PDT group and *P* = 0.018 in the ICGA-guided group) with no significant difference between the two groups (*P* = 0.10) [Table 2]. No cases with persistent SRF were found.

Throughout the follow-up period, none of the patients experienced any serious adverse events, including more than two lines of loss of BCVA, macular hemorrhage, or secondary CNV.

**Table 1:** Baseline characteristics of fluorescein and indocyanine green angiography-guided half-dose photodynamic therapy (PDT) groups.


	**FA (** ***n*** ** = 41)**	**ICGA (** ***n*** ** = 41)**	***P*** **-value**
Age (yr)	41.44 ± 7.61	41.56 ± 8.77	0.996 ${\;}^{*}$
Sex (male/female)	31/10	31/10	1.000 ${\;}^{**}$
Fovea involvement (yes/no)	28/13	32/9	0.455 ${\;}^{**}$
Duration of symptoms (months)	6.42 ± 8.80	18.14 ± 24.05	0.033 ${\;}^{*}$
Baseline BCVA (logMAR)	0.21 ± 0.19	0.20 ± 0.21	0.764 ${\;}^{*}$
Baseline CST (µm)	375.33 ± 98.69	385.63 ± 140.73	0.876 ${\;}^{*}$
Baseline SCT (µm)	456.27 ± 124.85	524.25 ± 138.78	0.205 ${\;}^{*}$
PDT spot size (µm)	3397.56 ± 1557.00 (1500-8000)	3387.80 ± 1636.18 (1600-8200)	0.966 ${\;}^{*}$
PDT spot numbers	1.39 ± 0.59 (1-3)	1.37 ± 0.49 (1-2)	0.951 ${\;}^{*}$
	
	
BCVA, best-corrected visual acuity; logMAR, logarithm of the minimum angle of resolution; CST, central subfield thickness; SCT, subfoveal choroidal thickness ${\;}^{*}$ Mann–Whitney U test; ${\;}^{**}$ Fisher's exact test			

**Table 2:** Visual and anatomic changes after half-dose photodynamic therapy (PDT) in fluorescein angiography-guided and indocyanine green angiography-guided groups.


	**FA**		P*	**ICGA**		P*	P**
	**Baseline**	**Last follow-up**	**Baseline**	**Last follow-up**	
BCVA (logMAR)	0.21 ± 0.19	0.07 ± 0.13	< 0.001	0.20 ± 0.21	0.06 ± 0.17	< 0.001	0.700
CST (µm)	375.33 ± 98.69	235.73 ± 31.40	< 0.001	385.63 ± 140.73	235.59 ± 33.22	< 0.001	0.564
SCT (µm)	456.27 ± 124.85	377.63 ± 103.13	< 0.001	524.25 ± 138.78	389.57 ± 120.62	0.018	0.108
	
	
BCVA, best-corrected visual acuity; logMAR, logarithm of the minimum angle of resolution; CST, central subfield thickness; SCT, subfoveal choroidal thickness ${\;}^{*}$ Wilcoxon signed-rank test; ${\;}^{**}$ Repeated Measure Analysis							

##  DISCUSSION

In CSC, the proposed mechanisms of action of PDT are temporary choriocapillaris occlusion and choroidal vascular remodeling, decreased choroidal vascular hyperpermeability, and resorption of SRF.^[5]^ Choriocapillaris occlusion and RPE ischemia with the subsequent complications, including secondary CNV have been reported as vision-threatening side effects with full-dose or standard PDT protocols.^[12]^ Therefore, alternative PDT protocols are suggested for the treatment of chronic CSC. These protocols include half-time PDT, half-dose PDT, and half-fluence PDT. Previous studies compared the application of these protocols and revealed their similar efficacy.^[13,14]^ In our study, FA-guided half-dose PDT provided a significant improvement in BCVA and a significant decrease in CMT and SCT, similar to that of the ICGA-guided half-dose PDT. These results were comparable with the results of similar previous studies.^[10,11]^

In most previous studies, ICGA guidance was used to determine the diameter of the PDT laser beam for the treatment of CSC.^[15,16]^ However, areas of choroidal vascular hyperpermeability may also be presented on ICGA images of asymptomatic fellow eyes of patients with CSC.^[17]^ Therefore, these hyper-permeable areas are not definitive indicators of clinically active disease and do not need treatment. Relying on areas of leakage in FA imaging may lead to smaller laser spots and possible foveal sparing in comparison to ICGA-guided PDT.^[10]^ In previous studies, the spot size in the FA-guided PDT group was significantly smaller than the ICGA-guided PDT group. This is in contrast to the result of our study, where there was no significant difference in laser spot size found between the two groups.^[10,11]^

In this study, a half-dose PDT approach was used for treatment. In previous studies with ICGA-guided half-dose PDT in chronic CSC, the rates of complete SRF resorption varied between 86.2 and 89.6%.^[13,18,19]^ Regarding the FA-guided half-dose PDT studies, the rates were 75.0–90.6%.^[14,20]^ Possible explanations for the differences in the rate of persistence of SRF after PDT among different studies may be the differences in the sample size, previous ocular history of participants, CSC characteristics, or the laser device used and varied protocols applied (time, dose, and fluence) for PDT. Previous studies suggest re-PDT for patients with recurrent or persistent SRF.^[10,11][14]^ In this study, we showed that in patients with persistent SRF, the fluid was resorbed completely and without any adverse sequences. Therefore, considering a decision for repeated PDT in refractory cases of chronic CSC must be done after assessing the inherent CSC characteristics and possible complications associated with intervention repetition.

In a study by Hayashida et al, the investigators compared visual and anatomical outcomes of FA-guided versus ICGA-guided half-time PDT in 61 eyes with chronic CSC.^[11]^ They found that both approaches had effective functional and anatomical outcomes for treating chronic CSC. However, they also found that the rate of persistence of SRF during a one-year follow-up was higher in the FA-guided group in comparison to the ICGA-guided group. They hypothesized that the lower rate of complete SRF resorption in patients with FA-guided PDT was possibly due to incomplete coverage of choroidal vascular hyperpermeability areas. Their study was limited by a less popular PDT technique (half-time) and the small number of eyes. In our study, both FA-guided and ICGA-guided half-dose PDT were efficient functionally and anatomically.

In a recent prospective study,^[21]^ half-dose PDT was compared between 24 patients in the ICGA-guided group and 12 in the FA-guided group. The ICGA-guided group had a significantly higher average total energy dose and spot count than the FA-guided group. The FA-guided group had smaller spot sizes and fewer spots. These results differ from our findings, which may be explained by the variations in the specialists' practice or sample size. Notably, the two groups had no differences in SCT, BCVA, or CST, which is in line with our results.

This study had some limitations. Firstly, the sample size was relatively small. Secondly, the follow-up time was short, and most of the patients were followed just until the time of complete SRF resorption. Therefore, probable recurrent cases were not reported. Thirdly, the cases were not prospectively randomized, and the FA-guided or ICGA-guided treatment was determined at the physicians' discretion. Further prospective controlled studies with a larger sample size and longer follow-up periods are required to make a better evaluation of the outcomes of different imaging modalities (FA vs ICGA) guidance for PDT in patients with CSC.

In summary, both FA- and ICGA-guided half-dose PDT may have similar efficacy for the treatment of CSC, and FA-guided half-dose PDT can be considered an alternative option for the treatment of CSC.

##  Financial Support and Sponsorship

None.

##  Conflicts of Interest

None.
